# Mental Health Mobile Apps in the French App Store: Assessment Study of Functionality and Quality

**DOI:** 10.2196/41282

**Published:** 2022-10-12

**Authors:** Florence Carrouel, Benjamin du Sartz de Vigneulles, Denis Bourgeois, Bernard Kabuth, Nicolas Baltenneck, Fanny Nusbaum, Valérie Burge, Sylvain Roy, Sophie Buchheit, Marie-Line Carrion-Martinaud, Catherine Massoubre, Laurie Fraticelli, Claude Dussart

**Affiliations:** 1 Health Systemic Process, Research Unit UR4129 University Claude Bernard Lyon 1 University of Lyon Lyon France; 2 Hospices Civils de Lyon Lyon France; 3 Prisme Team, Interpsy Laboratory, Research Unit EA4432, University of Lorraine Nancy France; 4 Nancy Psychotherapeutic Center Laxou France; 5 Development, Individual, Process, Disability, University Lyon 2 Lyon France; 6 The Constellation, Health and Wellness Center Rive-de-Gier France; 7 Department of Psychiatry, Research Unit EA7423, Saint-Etienne University Hospital Center of Saint Etienne, University Jean Monnet Saint Etienne France

**Keywords:** mobile apps, behavior change, mental, prevention, mobile health, mHealth, lifestyle, French, well-being

## Abstract

**Background:**

Approximately 800 million people, representing 11% of the world’s population, are affected by mental health problems. The COVID-19 pandemic exacerbated problems and triggered a decline in well-being, with drastic increase in the incidence of conditions such as anxiety, depression, and stress. Approximately 20,000 mental health apps are listed in mobile app stores. However, no significant evaluation of mental health apps in French, spoken by approximately 300 million people, has been identified in the literature yet.

**Objective:**

This study aims to review the mental health mobile apps currently available on the French Apple App Store and Google Play Store and to evaluate their quality using Mobile App Rating Scale–French (MARS-F).

**Methods:**

Screening of mental health apps was conducted from June 10, 2022, to June 17, 2022, on the French Apple App Store and Google Play Store. A shortlist of 12 apps was identified using the criteria of selection and assessed using MARS-F by 9 mental health professionals. Intraclass correlation was used to evaluate interrater agreement. Mean (SD) scores and their distributions for each section and item were calculated.

**Results:**

The highest scores for MARS-F quality were obtained by *Soutien psy avec Mon Sherpa* (mean 3.85, SD 0.48), Evoluno (mean 3.54, SD 0.72), and Teale (mean 3.53, SD 0.87). Mean engagement scores (section A) ranged from 2.33 (SD 0.69) for *Reflexe reussite* to 3.80 (SD 0.61) for *Soutien psy avec Mon Sherpa*. Mean aesthetics scores (section C) ranged from 2.52 (SD 0.62) for Mental Booster to 3.89 (SD 0.69) for *Soutien psy avec Mon Sherpa*. Mean information scores (section D) ranged from 2.00 (SD 0.75) for Mental Booster to 3.46 (SD 0.77) for *Soutien psy avec Mon Sherpa*. Mean Mobile App Rating Scale subjective quality (section E) score varied from 1.22 (SD 0.26) for *VOS – journal de l’humeur* to 2.69 (SD 0.84) for *Soutien psy avec Mon Sherpa*. Mean app specificity (section F) score varied from 1.56 (SD 0.97) for Mental Booster to 3.31 (SD 1.22) for Evoluno. For all the mental health apps studied, except *Soutien psy avec Mon Sherpa* (11/12, 92%), the subjective quality score was always lower than the app specificity score, which was always lower than the MARS-F quality score, and that was lower than the rating score from the iPhone Operating System or Android app stores.

**Conclusions:**

Mental health professionals assessed that, despite the lack of scientific evidence, the mental health mobile apps available on the French Apple App Store and Google Play Store were of good quality. However, they are reluctant to use them in their professional practice. Additional investigations are needed to assess their compliance with recommendations and their long-term impact on users.

## Introduction

Approximately 800 million people, representing 11% of the world’s population, are affected by mental health problems [1]. In addition, the COVID-19 pandemic exacerbated problems and triggered a decline in well-being, with a drastic increase in the incidence of conditions such as anxiety, depression, and stress [2]. However, within the World Health Organization’s classification of mental, behavioral, and neurodevelopmental disorders (International Classification of Diseases–11), a broad spectrum of mental conditions exists, varying from mild, one-time disorders to severe, chronic, and disabling disorders [3]. Similarly, the mental well-being concept encompasses more than the absence of mental health disorders and symptoms and may include psychological parameters such as subjective autonomy, well-being, personal fulfillment, and positive relationships [4]. There is growing recognition that mental health and mental well-being are distinct entities with specific determinants [5]. Preserving positive identity, maintaining good self-esteem, being able to control and adapt to one’s own life, and preventing social isolation and solitude are all positive aspects of well-being that can contribute to optimizing autonomy [6,7].

Mental disorders are a real public health problem [8]. Each year, approximately 20% of adults are affected by anxiety disorders [9]. Depression affects >300 million people worldwide [10,11]. In France, the mental health of French adults deteriorated between 2015 and 2020, partly because of the COVID-19 crisis, as indicated by a longitudinal study of adults [12]. French adults were more affected by depressive or anxiety symptoms. In addition, in recent years, the number of workers reporting complaints of severe stress has increased. Thus, stress is one of the most common occupational health problems. In Europe, the prevalence of men and women reporting work-related stress *always* or *most of the time* is 26% and 27%, respectively [13]. Stress at an early stage of working life can contribute to burnout, depression, and unfavorable work outcomes in later life, depending on the life course perspective [14,15]. Various psychosocial interventions have been suggested to overcome stress, such as relaxation, mindfulness practices, or social engagement, ideally performed in groups [16,17].

New technologies have the potential to overcome these challenges by providing large-scale health literacy programs, low-threshold approaches [18], or education for the population and health care workers [19]. In this sense, disruptive technologies provide a fantastic opportunity for enhancing mental health approaches [20]. Beyond supporting people with mental disorders, apps can also be used to improve their overall well-being by encouraging behavior changes, including the practice of meditation and mindfulness. Mobile apps related to mental health are known as mental health apps and vary in type and number [21]. In 2022, global spending on mental health mobile apps is estimated, not including China, to be approximately US $500 million [22]. Currently, there are reportedly up to 20,000 mental health apps [22].

Growing digital device use for mental health support also suggests mental health apps as a possible pertinent aspect for a proactive mental health and wellness model in the coming years [23]. Apps that capture the user’s moods or emotional conditions and then deliver information have significant and positive support, as do apps offering regular, short meditation sessions [24]. Mental health apps promote positive mental health and well-being, involving a decrease in symptoms of mental health conditions such as anxiety, stress, and depression. Mental health apps have the potential to be effective in improving the symptoms of certain mental disorders [25] and to improve well-being [26] and life satisfaction, with a more effective emotional management [21,24]. During the first 10 months of 2020, consumers spent a record of US $1.1 billion on wellness apps worldwide [22].

Nevertheless, no exploratory studies, which identify the development and application trends of evidence-based apps on topics such as outcomes of mental health or well-being apps, are currently available [25]. Further comprehensive validation and evaluation of these apps from a clinical perspective is needed [27].

The study aimed to review the mental health mobile apps currently available on the French Apple App Store and Google Play Store and to evaluate their quality using Mobile App Rating Scale–French (MARS-F).

## Methods

### Selection of the French Mobile Health Apps

Mental health–related apps were screened in the French App Store (iPhone Operating System [iOS]) and in the French Google Play Store (Android) from June 10, 2022, to June 17, 2022, by 2 academic researchers. The following search terms were used: “bien être mental” (mental well-being), “santé mentale” (mental health), and “bien être” (well-being). Each search term was introduced separately in the Apple App Store and Google Play Store because no truncation or use of logic operators (AND, OR, and NOT) was possible.

Each researcher eliminated duplicate apps by cross-checking the names of apps and developers. Then, both researchers checked that they had the same list of apps and downloaded them. They checked the following inclusion criteria: (1) mainly in the French language, (2) mental well-being as subject matter, (3) targeting adult users, and (4) self-personalized programs. The exclusion criteria were apps (1) focusing on content unrelated to mental health services, such as mental training, yoga, physical activity, and nutrition; (2) targeting people with specific disorders such as suicidal tendencies, eating disorders, or addiction; and (3) providing a single function only.

### Selection of Mental Health Professionals

The inclusion criteria for raters were the following: (1) mental health professional and (2) practicing at a hospital or performing private clinical activity in France. The exclusion criteria were (1) not having a mobile phone; (2) not being able to download apps from the Apple or Google stores; (3) never having used a mobile app; and (4) having hearing, visual, or motor disabilities.

### Selection of a Standardized Rating Scale for Mobile Apps

MARS-F [28] was used in this study. The first part of this scale, named *App classification*, includes the main characteristics of the app, such as the name, version, developer, focus or target, theoretical background or strategies, age group, and so on. This part was reviewed by the 2 academic researchers. The Mobile App Rating Scale (MARS) scale is composed of a main part (23 items organized into 5 sections, named A, B, C, D, and E) and an additional part (section F with 6 items).

Section A (engagement section; 5 items) determines whether the app is interesting, fun, customizable, and interactive (sends alerts, feedback, reminders, and messages and allows sharing). Section B (functionality section; 4 items) analyzes the app operation, ease of learning, flow logic, navigation, and gestural design of the app. Section C (aesthetics section; 3 items) focuses on the graphic design of the app, color palette, overall visual appeal, and stylistic consistency. Section D (information quality; 7 items) assesses whether the app contains high-quality information (feedback, text, references, and measurements) from a credible source. Section E (subjective section; 4 items) determines the interest of the user in the app. Section F (mobile app specificities; 6 items) analyzes the point of view of mental health professionals regarding the effect of selected apps on knowledge, possible changes in user attitudes and intentions to change, and probability of changing the identified targeted behaviors. In our study, the targeted health behavior was *mental well-being*.

Each item was rated on a 5-point Likert scale (1=strongly disagree to 5=strongly agree). The score for each section was obtained by calculating the mean score of the items. The mean of section scores (sections A, B, C, and D) corresponds to the overall quality MARS score. The mean score of section E corresponds to the subjective quality score, and the mean score of section F evaluates the specificities of the app. The scores range from a minimum of 1 (poor quality) to a maximum of 5 (high quality).

### Methodology of Evaluation

#### Training of the Raters

The raters were 9 mental health professionals (Multimedia Appendix 1). Before rating the mental well-being apps, the raters had to train in the use of MARS-F. For this, they viewed a training video in French (available on request to the corresponding author), which was developed for MARS-F [28] and adapted from the English training video of Stoyanov et al [29]. In this video, each item and answer is explained based on examples. At the end of the video, a training exercise with an app not included in the sample of the study was proposed. The raters downloaded the app, tested it for at least 10 minutes, completed MARS-F, and then compared their results with those in the video. When the individual score for an item differed by >2 points, the raters discussed until a consensus was reached to ensure that they had the same understanding.

#### Evaluation of the Selected Apps by the Raters

The mental health apps were evaluated by the 9 mental health professionals during the month of July 2022. They downloaded all the included apps, used each app for at least 10 minutes, and immediately evaluated the app using a web-based MARS-F questionnaire.

### Statistical Analysis

To assess interrater reliability, the intraclass correlations (ICCs; 2-way random, average measures and absolute concordance) were calculated [30,31]. For each item, each section, and MARS-F quality score (sections A, B, C, and D), the 95% CIs were calculated. On the basis of the 95% CI of the ICC estimate, values <0.5, between 0.5 and 0.75, between 0.75 and 0.9, and >0.90 are indicative of poor, moderate, good, and excellent reliability, respectively [31]. Mean values and SDs were calculated for each item and each mental health app section (presented as mean [SD]). Owing to missing values, item 19 was excluded from all analyses, and the mean for section D was adjusted accordingly.

To assess the differences between the quality of the apps, by item and by section, box plots were generated. The points that appear on either side of the boxes are calculated using 1.5 times the IQR (the distance between the first and third quartiles) to highlight the extreme values.

To provide an overview of the average scores for each item (row) and each app (column), a heat map was constructed. The color gradients represent whether the score is low (near 1 [yellow]) or high (near 5 [green]).

To assess the correlation between average quality and subjective item 23 (“What is your overall star rating of the app?”), the Pearson coefficient (*r*) was calculated. To provide a complete overview of the popularity of each mobile app and the number of reviewers, the number of stars awarded by users in the iOS and Android stores was reported.

Statistical analyses were performed using R, with the *dplyr*, *psych*, and *ggplot2* packages from the R Project for Statistical Computing (version 4.1.1; R Foundation for Statistical Computing).

## Results

### Selection of Mental Health Mobile Apps

**Figure 1:**
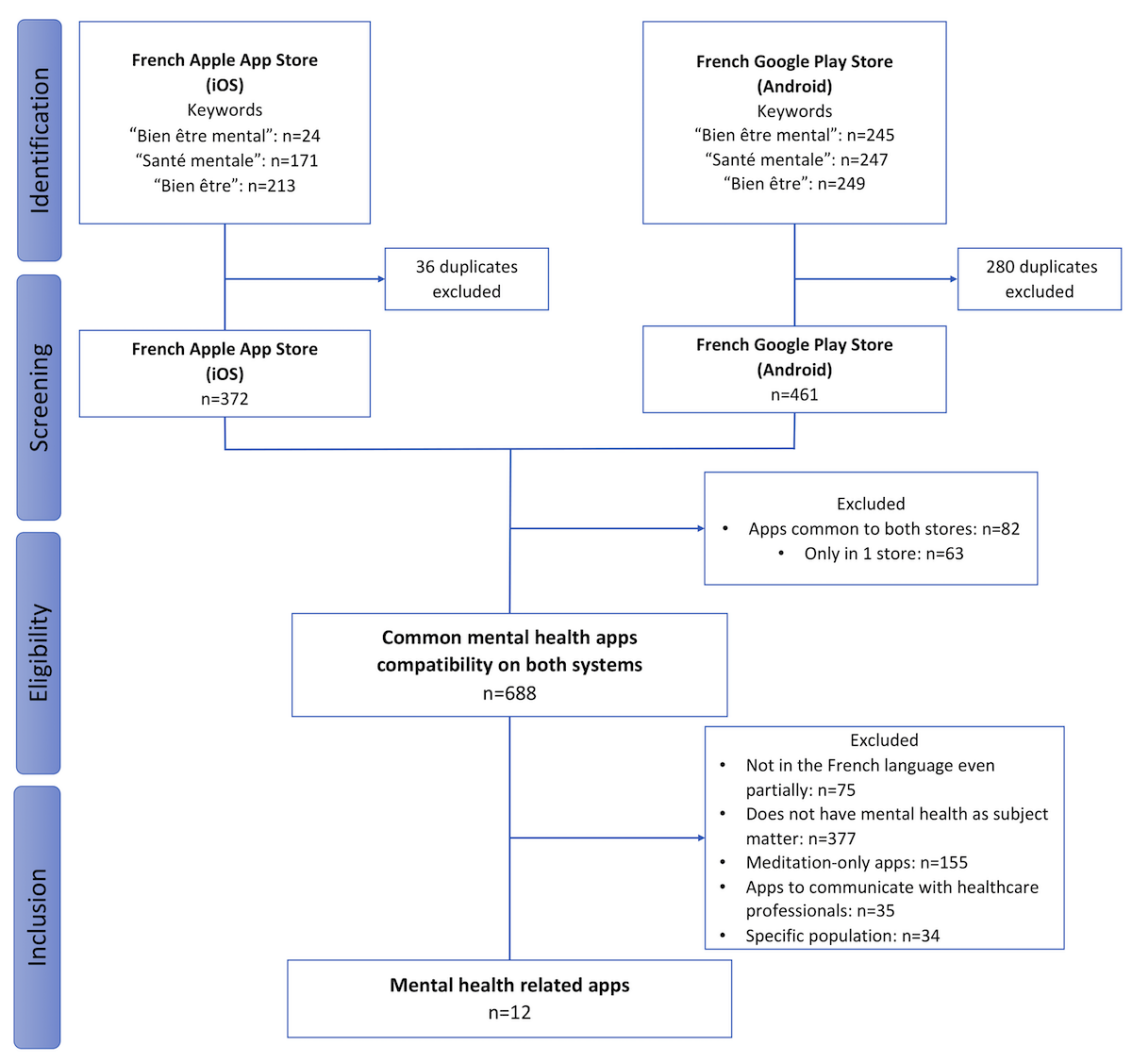
Flowchart of mental health mobile apps selection. iOS: iPhone Operating System.

The use of keywords allowed the identification of 35.51% (408/1149) of apps from Apple App Store and 64.49% (741/1149) of apps from Google Play Store (Figure 1). Once the 2 lists were cross-checked according to the name of the app and developer, 59.87% (688/1149) of the apps were common to both systems. After a thorough review of the remaining apps’ download page and application of the inclusion and exclusion criteria, 1.04% (12/1149) of the apps were finally selected.

### Characteristics of Mental Health Mobile Apps

Descriptive and technical information regarding the mobile mental health apps is provided in Tables S1-S6 in Multimedia Appendix 2. None of these apps had the same developer. An app had a different name depending on the store (*VOS* – *journal de l’humeur* in the Apple App Store and *VOS* – *journal intime* in the Google Play Store), whereas all the other apps had the same name. Of the 12 apps, 1 (8%) was fully free of charge, 8 (67%) were free with in-app purchases, and 3 (25%) were accessible exclusively with a company code. The number of downloads depends on the app. According to Google Play Store (as the data were not available for the Apple store), Sanvello was the most downloaded app, followed by *VOS* – *journal de l’humeur*.

Table 1 presents the characteristics of the mental health mobile apps. All the apps (12/12, 100%) targeted increasing happiness or well-being; mindfulness, meditation, or relaxation; negative emotion reduction; anxiety or stress; anger; behavior change; and goal setting, whereas only 17% (2/12) of the apps targeted alcoholism or substance use, 17% (2/12) focused on relationships, and another 17% (2/12) aimed at physical health. The theoretical background and strategies of all apps were the following: assessment; information or education; monitoring or tracking; goal setting; advice, tips, strategies, or skills training; cognitive behavioral therapy (CBT)—behavioral (positive events); CBT—cognitive (thought challenging); acceptance commitment therapy; and mindfulness or meditation. Most mental health apps (11/12, 92%) also used monitoring or tracking as strategies. The apps were designed for young adults (12/12, 100%) and adults (12/12, 100%) but not for adolescents and children (aged <12 years). All the mental health apps (12/12, 100%) allowed password protection, required log-in, sent reminders, and needed web access to function, and 67% (10/15) of the apps required web access to function. Of the 12 apps, 6 (50%) allowed sharing (Facebook, Twitter, etc) and 5 (42%) had an app community.

**Table 1:** Characteristics of the mental health mobile apps (N=12).

Characteristics		Apps, n (%)
**Focus—what the app targets^a^**		
	Increase happiness or well-being	12 (100)
	Mindfulness, meditation, or relaxation	12 (100)
	Reduce negative emotions	12 (100)
	Anxiety or stress	12 (100)
	Anger	12 (100)
	Behavior change	12 (100)
	Alcohol or substance use	2 (17)
	Goal setting	12 (100)
	Relationships	2 (17)
	Physical health	2 (17)
**Theoretical background or strategies^a^**		
	Assessment	12 (100)
	Information or education	12 (100)
	Monitoring or tracking	11 (92)
	Goal setting	12 (100)
	Advice, tips, strategies, or skills training	12 (100)
	CBT^b^—behavioral (positive events)	12 (100)
	CBT—cognitive (thought challenging)	12 (100)
	Acceptance commitment therapy	12 (100)
	Mindfulness or meditation	12 (100)
	Relaxation	12 (100)
**Age group^a^**		
	Children (<12 years)	0 (0)
	Adolescents (13-17 years)	0 (0)
	Young adults (18-25 years)	12 (100)
	Adults (>25 years)	12 (100)
**Technical aspects of the app^a^**		
	Allows sharing (Facebook, Twitter, etc)	6 (50)
	Has an app community	5 (42)
	Allows password protection	12 (100)
	Requires log-in	12 (100)
	Sends reminders	12 (100)
	Needs web access to function	12 (100)

^a^Participants could choose several answers.

^b^CBT: cognitive behavioral therapy.

### Reliability of the Evaluation

The ICC, calculated based on the 12 apps, was considered as good, with 0.79 (95% CI 0.73-0.83) for the A, B, C, and D sections. The ICC per section was good for section A (0.68, 95% CI 0.54-0.79), section B (0.62, 95% CI 0.44-0.76), section C (0.63, 95% CI 0.42-0.78), and section D (0.81, 95% CI 0.73-0.87).

### Assessing the Quality of Content of Mental Health Mobile Apps

**Figure 2:**
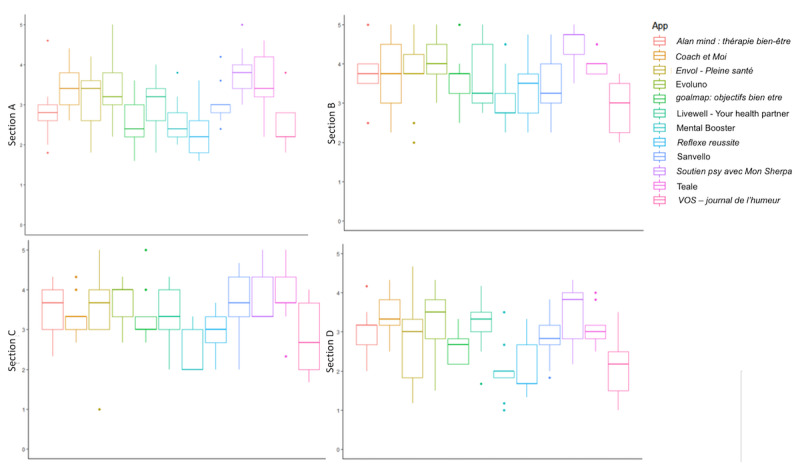
Qualitative evaluation of mental health mobile apps—section A: engagement, section B: functionality, section C: esthetics, and section D: information.

MARS-F quality score for each section (sections A, B, C, and D) and each app are presented in Figure 2 and Tables S1 and S2 in Multimedia Appendix 3. Mean engagement scores (section A) ranged from 2.33 (SD 0.69) for *Reflexe reussite* to 3.80 (SD 0.61) for *Soutien psy avec Mon Sherpa*. Mean functionality scores (section B) ranged from 2.89 (SD 0.63) for *VOS* – *journal de l’humeur* to 4.47 (SD 0.53) for *Soutien psy avec Mon Sherpa*. Mean esthetics scores (section C) ranged from 2.52 (SD 0.62) for Mental Booster to 3.89 (SD 0.69) for *Soutien psy avec Mon Sherpa*. Mean information scores (section D) ranged from 2 (SD 0.75) for Mental Booster to 3.46 (SD 0.77) for *Soutien psy avec Mon Sherpa*. For all mental health apps, except Sanvello (11/12, 92%), the mean score for functionality was consistently higher than that for the other sections.

**Figure 3:**
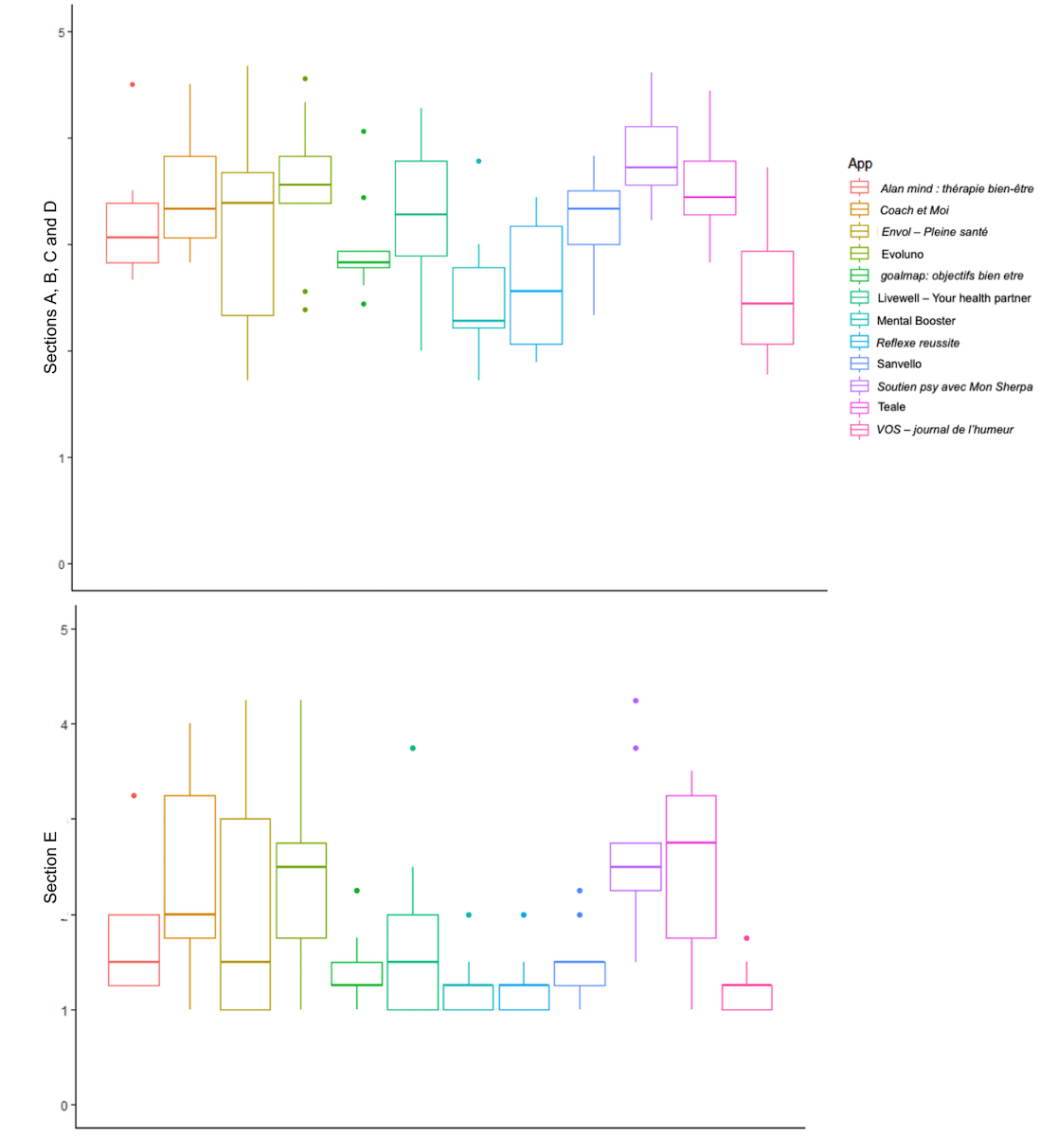
Qualitative (sections A, B, C, and D) and subjective qualitative (section E) evaluation of mental health mobile apps.

MARS-F quality score (sections A, B, C, and D) and MARS-F subjective quality scores (section E) for each app are presented in Figure 3 and Table S1 in Multimedia Appendix 3. The best MARS-F quality scores were obtained by *Soutien psy avec Mon Sherpa* (mean 3.85, SD 0.48), Evoluno (mean 3.54, SD 0.72), and Teale (mean 3.53, SD 0.47), whereas the worst quality scores were obtained by *Reflexe reussite* (mean 2.59, SD 0.61), *VOS – journal de l’humeur* (mean 2.55, SD 0.71), and Mental Booster (mean 2.49, SD 0.61). Mean MARS-F subjective quality score varied from 1.22 (SD 0.26) for *VOS – journal de l’humeur* to 2.69 (SD 0.84) for *Soutien psy avec Mon Sherpa*. The best subjective quality scores were obtained by *Soutien psy avec Mon Sherpa* (mean 2.69, SD 0.84), Teale (mean 2.53, SD 0.91), and Evoluno (mean 2.42, SD 1), whereas the worst quality scores were obtained by Mental Booster (mean 1.25, SD 0.33), *Reflexe reussite* (mean 1.25, SD 0.33), and *VOS – journal de l’humeur* (mean 1.22, SD 0.26).

### Assessing the Content Specificity of Mental Health Mobile Apps

**Figure 4:**
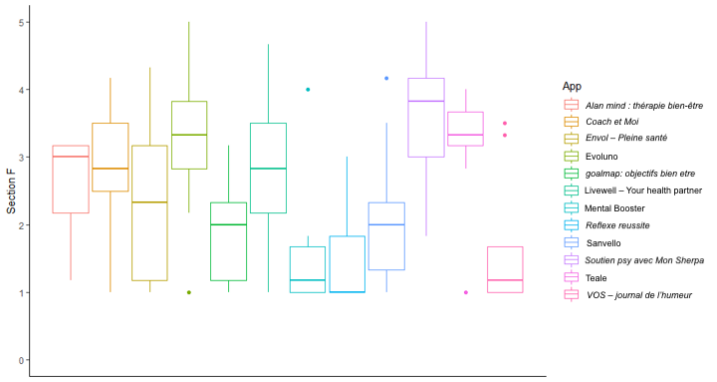
App-specific scores of mental health mobile apps (section F).

The evaluation of the specificity (section F) of the mental health apps is summarized in Figure 4 and Table S1 in Multimedia Appendix 3. This score (mean) ranged from 1.56 (SD 0.97) for Mental Booster to 3.31 (SD 1.22) for Evoluno. The best specificity scores were achieved by *Soutien psy avec Mon Sherpa* (mean 2.59, SD 0.92), Teale (mean 3.20, SD 0.90), and Evoluno (mean 3.31, SD 1.22), whereas the worst quality scores were obtained by Mental Booster (mean 1.56, SD 0.97), *Reflexe reussite* (mean 1.57, SD 0.76), and *VOS – journal de l’humeur* (mean 1.65, SD 1.02).

### Evaluation of the Strengths and Weaknesses of Each App

The app-specific score (section F) was always lower than the subjective quality score (section E), which was always lower than the MARS-F quality score (sections A, B, C, and D), except for *Soutien psy avec Mon Sherpa*. This score was lower than the rating from Apple App Store or Google Play Store, when the apps are rated in the stores (Multimedia Appendix 2).

**Figure 5:**
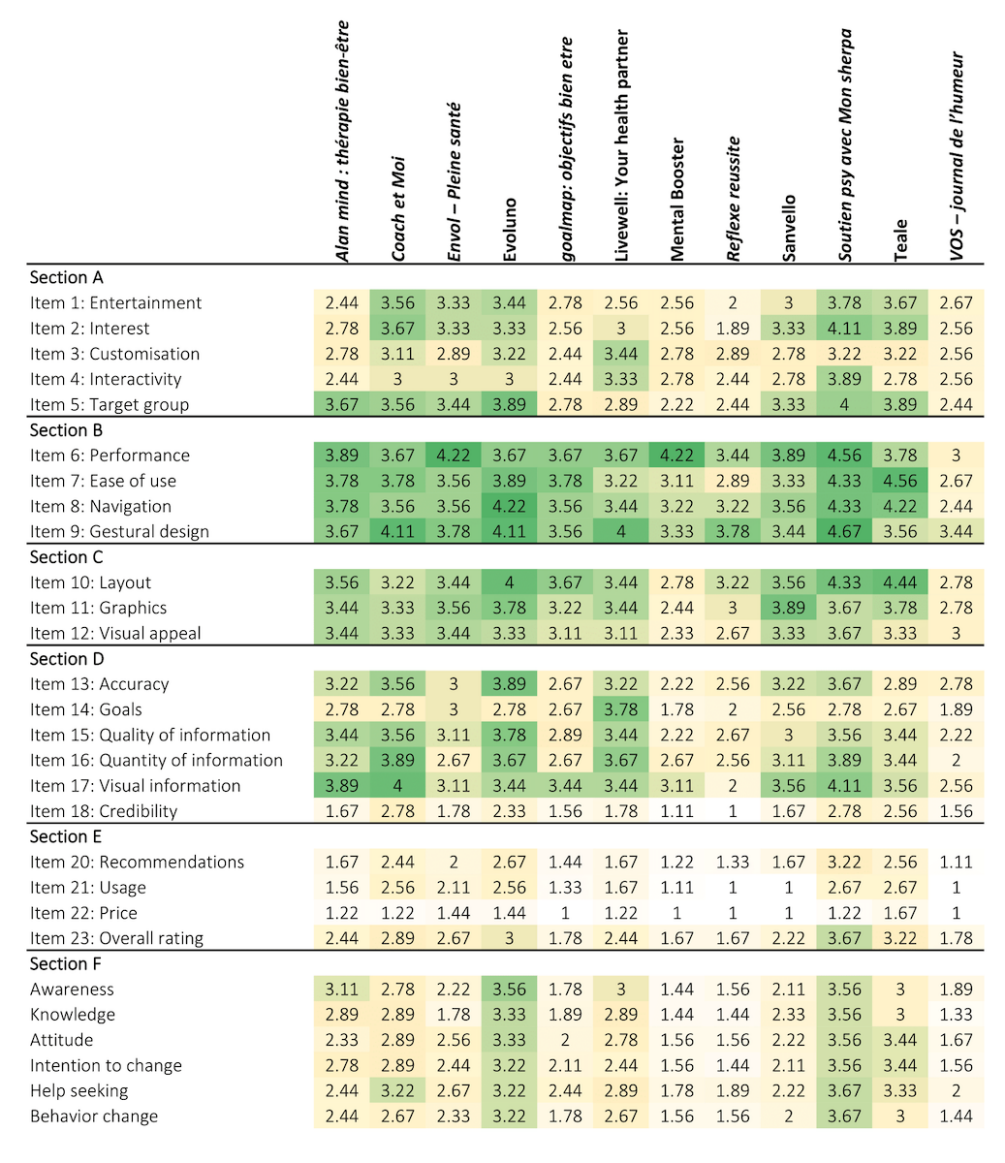
Heat map of the average scores for each item and each app. The colors are related to the scores and range from yellow (1=worst score) to green (5=best score).

The graphical comparison of the average scores for each item and each app (Table S2 in Multimedia Appendix 3) is shown in the heat map (Figure 5). The weakness of the quality score (sections A, B, C, and D) was mainly owing to sections A and D, except *for Coach et Moi*, Evoluno, Livewell - Your health partner, and *Soutien psy avec Mon Sherpa*. More particularly, in section D, the worst scores were observed for the credibility of the app (item 18), except for *Coach et Moi*, *Soutien psy avec Mon Sherpa*, and Teale, with mean 2.78 (SD 1.30), mean 2.78 (SD 0.83), and mean 2.56 (SD 0.73), respectively. The subjective quality (section E) was affected by the weak score regarding the price of the app (item 22). For the specificity of the apps (section F), no real weakness was observed because all the apps had similar item scores. It should be noted that Evoluno and *Soutien psy avec Mon Sherpa* obtained the highest scores.

### Correlation Between MARS-F Score and Star Rating

The correlation between the MARS-F quality score (sections A, B, C, and D) and MARS-F overall star rating (item 23: “What is your overall star rating of the app?”) was considered as good (*r*=0.86; *P*<.001). The MARS-F quality score was higher than the MARS-F overall star rating for all the mental health apps tested (12/12, 100%). Correlation analysis between the MARS-F quality score of the apps and their respective star rating in the mobile app stores was limited by the availability of star rating in the stores and discrepancies among the number of raters. The store ratings were high compared with the MARS-F quality score. These user ratings fluctuate from 2.7 (*VOS* – *journal de l’humeur*) to 4.8 (*Alan Mind: thérapie bien-être*) for the iOS store and from 3.2 (*Coach et Moi*) to 4.6 (*Envol - Pleine santé*) for the Android store (Table 2).

**Table 2:** MARS-F^a^ overall star rating, overall quality MARS-F score, star rating in the Apple App Store, and star rating in the Google Play Store.

Apps	MARS-F overall star rating (item 23), mean (SD)	MARS-F quality score (sections A, B, C, and D), mean (SD)	Apple App Store, star rating; number of raters	Google Play Store, star rating; number of raters
*Alan Mind: thérapie bien-être*	2.44 (0.53)	3.22 (0.56)	4.8; 3217	4.2; 125
*Coach et Moi*	2.89 (1.27)	3.47 (0.55)	4.6; 28	3.2; 32
*Envol - Pleine santé*	2.67 (1.41)	3.23 (0.97)	4.8; 30	4.6; 156
Evoluno	3.00 (1.22)	3.54 (0.72)	N/A^b^	N/A
*goalmap: objectifs bien etre*	1.78 (0.44)	2.97 (0.49)	4.2; 269	3.6; 2000
Livewell - Your health partner	2.44 (1.13)	3.28 (0.73)	N/A	N/A
Mental Booster	1.67 (0.71)	2.49 (0.61)	3.1; 11	N/A
*Reflexe reussite*	1.67 (0.71)	2.59 (0.61)	4.4; 10	4; 28
Sanvello	2.22 (1.09)	3.18 (0.54)	4.3; 339	4.2; 23,000
*Soutien psy avec Mon Sherpa*	3.67 (1.00)	3.85 (0.48)	4.6; 1409	4.3; 1000
Teale	3.22 (1.09)	3.53 (0.47)	4.2; 17	4.3; 14
*VOS* – *journal de l’humeur*	1.78 (0.83)	2.55 (0.71)	2.7; 14	4.4; 16,000

^a^MARS-F: Mobile App Rating Scale–French.

^b^N/A: not applicable.

## Discussion

### Principal Findings

Mental health apps aim to improve mental health and wellness, whether in terms of curing a mental illness or implementing habits to improve emotional health [32]. A public survey showed that 76% of the respondents would like to have access to free apps to self-manage and self-monitor their mental health [33]. Thus, mental health apps can play a critical role in mental health care [34]; making mental health care accessible; and reducing barriers to help-seeking, as few people with mood or anxiety problems seek professional help. In addition, geographic, financial, or social constraints may make it difficult to access support [21].

Mental health apps cover all stages of clinical care delivery, including prevention, diagnosis, primary treatment [35], and so on. Our study focused on mental health well-being apps that were consistent with health promotion. Thus, we included apps that enabled users’ mental health self-management and that did or did not offer appointments with mental health professionals. We excluded apps whose only purpose was to schedule appointments with mental health professionals, because their focus was more treatment-oriented. Screening of mental health apps available in the French iOS and Android stores led to the inclusion of 12 apps. In Singapore, screening of iTunes and Google Play Store led to inclusion of 44 mental health apps [36]. Compared with our study, they used more keywords, and several of them were related to COVID-19. In China, the screening permitted to select 40 apps, but the authors screened the apps in Apple App Store (for iOS apps), Tencent My App, and Huawei App Gallery (for Android apps), and they selected apps in the Chinese language [37]. In our 2 previous studies using the same methodology but focusing on the risk factors of noncommunicable diseases, 15 apps related to nutrition [38] and 9 apps related to oral hygiene [39] were included.

All the included apps aimed to increase happiness, well-being, mindfulness, meditation, and relaxation and reduce negative emotions, anxiety, stress, and anger. Some apps additionally targeted alcohol or substance use (Livewell - Your health partner and Teale), relationships (Livewell - Your health partner and *VOS* – *journal de l’humeur*), or physical activity (*goalmap: objectifs bien etre* and Livewell - Your health partner). These mental health apps mainly used CBT, acceptance commitment therapy, and mindfulness or mediation to reach their goals. The use of CBT in mental health apps was one of the recommendations of Bakker et al [21]. CBT is a form of collaborative, individualized psychological treatment that is recognized as the most sustained approach for generating behavioral, cognitive, and emotional adaptation to a wide range of common psychological problems [40]. Several studies have concluded that CBT is an effective treatment for a wide range of psychological disorders, which can be administered via a mobile device and still retain its therapeutic validity [21]. Moreover, CBT can also prevent psychological problems from becoming worse [41-43].

The MARS-F quality score was >2.5, for all the mental health apps tested, except for Mental Booster (11/12, 92%; mean 2.49, SD 0.61). Mean score ranged from 2.49 (SD 0.61) to 3.85 (SD 0.48). Similar scores were observed for apps related to nutrition available in France (mean 3.34, SD 0.39 to mean 3.84, SD 0.32) [38] and oral hygiene (mean 1.80, SD 0.79 to mean 3.4, SD 0.97) [39]. *Soutien psy avec Mon Sherpa,* which obtained the highest score (mean 3.85, SD 0.48), had the particularity of offering real-time interactions with an artificial intelligence–enabled chatbot to collect and record daily mood. Thus, the user can develop a relationship with the chatbot, similar to the one observed between a therapist and their patient, which involves collaborative empiricism [44]. The 2 apps (Evoluno: mean 3.54, SD 0.72 and Teale: mean 3.53, SD 0.47) that received the highest MARS-F scores had a different concept. They provided some information to the user (resilience, stress, anxiety, negative thoughts, etc) in the form of a podcast (Evoluno) or videos (Teale). Evoluno proposed to evaluate the user (sleep quality, risk of burnout, depression, etc) and suggested breathing exercises. Teale proposed to set goals for the user (self-control, self-esteem, self-fulfillment, etc) with various activities (meditation, breathing, writing, etc) to reach them. Moreover, these 2 apps permitted to contact mental health professionals.

The functionality (section B) was the strength for all the apps tested, except for Sanvello (11/12, 92%). Our previous studies demonstrated that it was also the strength of French nutrition–related and oral hygiene–related apps [38,39]. Information quality (section D) was the weakness of all the apps, except 4 apps (8/12, 67%; *Alan Mind: thérapie bien-être*, *Coach et Moi*, *goalmap: objectifs bien etre*, and Livewell - Your health partner). The weak point of Coach et Moi was the aesthetics (section C), which was not observed for any of the French apps related to nutrition or oral hygiene included in our previous studies [38,39]. The worst score in section D was observed for the credibility of the app because the mental health professionals identified the source of information, but they evaluated that its validity or reliability was questionable (eg, commercial enterprise with vested interest). In addition, as the level of scientific evidence is difficult to assess, mental health professionals chose “N/A The application has not been tested” in most cases to answer item 19, which therefore could not be included in the statistical analysis. To our knowledge, of the 12 apps, only 1 (8%) app included in this study (Sanvello) was indexed in PubMed [45-48]. Myers et al [45] evaluated apps for depression self-management, Mehdi et al [46] evaluated apps for Tinnitus, and Lau et al [47] evaluated apps for mental health. In these 3 studies, the observed MARS scores for Sanvello were 4.6, 4.6, and 4.28, respectively, which were higher than those observed in our study (mean 3.18, SD 0.54). This difference could be explained by the fact that, in these studies, the number of raters was 2 [45,47] or 4 [46] at most.

The MARS-F quality score (sections A, B, C, and D) was higher than the specificity of the app (section F), which was higher than the subjective quality (section E), except for *Soutien psy avec Mon Sherpa.* For this app, the specificity of app (section F) obtained the low score. The subjective quality score (section E) was >2.5 only for *Soutien psy avec Mon Sherpa* (mean 2.69, SD 0.84) and Teale (mean 2.53, SD 0.91). For all the apps tested (12/12, 100%), the main problem was the cost, because mental health professionals declared that they will not pay for this app. The specificity of the apps (section F) assessed the perceived impact of the app on the user’s knowledge, attitudes, intent to change, and likelihood of actual change regarding mental health. Only 50% (6/12) of the mental health apps included obtained a score >2.5 for this section. Evoluno (mean 3.31, SD 1.22), Teale (mean 3.20, SD 0.90), and *Coach et Moi* (mean 2.89, SD 0.95) obtained the best scores, followed by Livewell - Your health partner (mean 2.78, SD 1.12), *Alan Mind: thérapie bien-être* (mean 2.67, SD 0.72), and *Soutien psy avec Mon Sherpa* (mean 2.59, SD 0.92). These results can be explained because professionals preferred apps containing information that promotes mental health and well-being than a chatbot.

In contrast to previous studies on French nutrition–related [38] and oral hygiene–related [39] apps, the star rating score from the iOS and Android stores was always lower than the subjective quality score (section E). This difference may be explained by the fact that, when the mental health apps were evaluated in the stores, they were evaluated by a very small number of evaluators, which is in contrast to the nutrition and oral hygiene apps, which were evaluated by a large number of evaluators. The difference between the star rating score and subjective quality score was owing to the fact that the evaluations in the mobile app stores are made by all the users, whereas the evaluation in this study was made by mental health professionals using the MARS-F. Thus, it could be interesting to evaluate the same apps using the user version of the MARS (uMARS) that is designed for users to compare the results.

### Limitations

This study has several limitations. First, only mental health apps available on both French Apple App Store and Google Play Store were included, even though several other stores could be investigated (Huawei store, BlackBerry World, Windows Phone Store, or Samsung store). Second, regarding the protection of personal data, no criteria have been defined, except for password protection to log in to the apps. This criterion is not sufficient to prove that the user’s personal data are protected.

Third, the commercial use of mobile mental health apps was not considered as a key criterion for assessing the acceptability of the app to users when many mobile apps are developed by anonymous companies but funded by large groups such as pharmaceutical companies. Fourth, MARS was selected for this investigation because it is the most commonly used scale in scientific literature for the assessment of mental health apps [38,39,47,49-59], and its use has been recommended by the French High Authority of Health [60]. Nevertheless, other scales, such as ENLIGHT, which also assesses mobile health apps, could have been used [61]. Correlation and good parallel validity between the MARS and ENLIGHT have been shown [62]. Fifth, our study is only applicable to the French-speaking community, where these mobile apps are available. Sixth, the evaluation was conducted by mental health professionals, whereas the mobile health apps were developed for the general population. Therefore, it will be interesting to have these apps evaluated by users, using the uMARS scale [63] that was developed for them, and then compare the results.

### Perspectives

The COVID-19 pandemic has changed the use of digital technology in the field of mental health and, more particularly, by mental health professionals. The pandemic has made it possible to revisit the ways in which the most fragile people are cared for with connected tools and to move toward an evolution of practices [64]. Mental health apps can improve accessibility to mental health services and be beneficial for mental health [32]. They have positive effects on depressive symptoms, anxiety, and other mental health problems [65] and help overcome some of the barriers that make it difficult to access mental health services (stigma, cost, capacity, and geography) [66]. Thus, this study can help mental health professionals and mobile app users to choose the best mental health apps available in France, in terms of quality.

Health care professionals generally have a different perspective than users. Thus, it may be interesting to have the mental health apps studied in this study evaluated by users via the uMARS scale and then compared.

This study qualitatively assessed mental health–related apps but did not focus on content analysis of the included apps. A review of the literature specific to apps supporting mental health and wellness concluded that apps for bipolar disorder were cost-effective and convenient but that most of them did not provide information on all the core principles of psychoeducation and did not adhere to best practice guidelines [67]. Similarly, Larsen et al [68] showed that many suicide prevention apps were not evidence-based and determined that some apps may be more harmful than helpful. They found that some apps included potentially extremely harmful content describing or supporting access to lethal means, encouraging people to end their lives, and presenting suicide in a trendy manner [66]. Moreover, as many apps are developed for commercial purposes, ethical questions arise regarding the use of personal data. Therefore, additional studies analyzing the content of the apps are needed.

Furthermore, the implementation of randomized clinical trials or longitudinal studies including the 12 mental health apps selected in this study should allow for the analysis of the change in mental health status after the use of the apps.

### Conclusions

Mental health professionals assessed that, despite the lack of scientific evidence, the mental health mobile apps available on the French Apple App Store and Google Play Store were of good quality. However, they are reluctant to use them in their professional practice. Additional investigations are needed to assess their compliance with recommendations and their long-term impact on users.

## Appendix

Multimedia Appendix 1 Characteristics of the raters and the hardware and software used.

Multimedia Appendix 2 Descriptive and technical information about the mental health mobile apps.

Multimedia Appendix 3 Mobile App Rating Scale scoring.

## Abbreviations

CBT: cognitive behavioral therapy
ICC: intraclass correlation
iOS: iPhone Operating System
MARS: Mobile App Rating Scale
MARS-F: Mobile App Rating Scale–French
uMARS: user version of the Mobile App Rating Scale
