# Supplementary material for: Mental Health Mobile Apps in the French App Store: Assessment Study of Functionality and Quality
Source: JMIR Mhealth Uhealth. 2022 Oct 12;10(10):e41282. doi: 10.2196/41282 (PMC9607929; doi:10.2196/41282)
Supplement: Multimedia Appendix 1 [file mhealth_v10i10e41282_app1.docx]

**Multimedia Appendix 1:**

**Characteristics of the raters, the hardware and the software used.**

|  | **Rater 1** | **Rater 2** | **Rater 3** | **Rater 4** | **Rater 5** | **Rater 6** | **Rater 7** | **Rater 8** | **Rater 9** |
| --- | --- | --- | --- | --- | --- | --- | --- | --- | --- |
| **Age** | 54 | 46 | 40 | 51 | 37 | 38 | 44 | 44 | 61 |
| **Year of experience in mental health** | 22 | 20 | 18 | 27 | 18 | 5 | 14 | 17 | 32 |
| **Affiliation** | Private activity | Private activity | Hospital | Hospital | Hospital | Private activity | Hospital | Hospital | Private activity |
| **Scope of activity** | Clinical psychologist | Clinical psychologist | Clinical psychologist | Clinical psychologist | Nurse in psychiatric emergencies and in medical-psychological centers | Clinical psychologist | Nurse in medical-psychological center | Clinical psychologist | psychiatrist |
| **Hardware (phone)** | Samsung | iPhone | Huawei | Huawei | Xiaomi | iPhone | Xiaomi | iPhone | Samsung |
| **Software (iOS or Android)** | Android | iOS | Android | Android | Android | iOS | Android | iOS | Android |
