# Supplementary material for: Mental Health Mobile Apps in the French App Store: Assessment Study of Functionality and Quality
Source: JMIR Mhealth Uhealth. 2022 Oct 12;10(10):e41282. doi: 10.2196/41282 (PMC9607929; doi:10.2196/41282)
Supplement: Multimedia Appendix 2 [file mhealth_v10i10e41282_app2.docx]

**Multimedia Appendix 2:**

**Descriptive and technical information of the mental health mobile apps**

**Table S1.** List of the 12 mental health mobile apps included in the study and their scare rating in iOS and Android stores

| **App name** | **Developper** | **Rating in the iOS app store**  **(Nb of raters)** | **Rating in the Android app store**  **(Nb of raters / Nb of downloads)** | **Paid Content** |
| --- | --- | --- | --- | --- |
| *Alan Mind: thérapie bien-être* | Alan Health | 4.8  (3152) | 4.2  (125 / > 5 000) | Free with in-app purchases |
| *Coach et Moi* | Malakoff Humanis | 4.6  (28) | 3.2  (32 / > 5 000) | Free with in-app purchases |
| *Envol – Pleine santé* | Envol | 4.8  (30) | 4.6  (156 / > 10 000) | Free with in-app purchases |
| Evoluno | Evoluno | NA  (NA) | NA  (NA / > 1 000) | Access with a company code |
| *goalmap: objectifs bien etre* | goalmap | 4.2  (269) | 3.6  (2 000/ > 100 000) | Free with in-app purchases |
| LiveWell – Your health partner | Zurich Insurance Company Ltd | NA  (NA) | NA  (NA / > 5 000) | Access with a company code |
| *Reflexe reussite* | PPI | 4.4  (10) | NA  (NA / > 100) | Free with in-app purchases |
| Mental Booster | Olivier Madelrieux | 3.1  (11) | 4.0  (28 / 1 000) | Free with in-app purchases |
| Sanvello | Sanvello Health Inc. | 4.3  (339) | 4.2  (23 000 / > 1 M) | Free with in-app purchases |
| *Soutien psy avec Mon Sherpa* | doctoconsult | 4.6  (1408) | 4.3  (1000 / > 100 000) | Free |
| Teale | ENSOPCO | 4.2  (16) | 4.3  (14 / > 1 000) | Access with a company code |
| *VOS – journal de l'humeur* | Vos | 2.7  (14) | 4.4  (16 000 / > 500 000) | Free with in-app purchases |

**Table S2.** Brief description of the 12 mental health mobile apps included in the study

| **App name** | **Brief description of apps** |
| --- | --- |
| *Alan Mind: thérapie bien-être* | This app makes the practice of journaling and Cognitive Behavioral Therapy simple and accessible to all through interactive guides designed to meet your needs (developing self-esteem, humility and gratitude, practicing mindfulness, achieving your goals but also identifying and fighting your anxieties or loneliness). It allows you to talk to specialists |
| *Coach et Moi* | This app allows the user to measure stress level and identify its causes, to create a personalized program based on meditation and yoga, and to discuss with a clinical psychologist. |
| *Envol – Pleine santé* | This app allows the user to calculate a well-being score and a balance between activity and rest. Thanks to its algorithm, this app sends personalized texts to improve lifestyle habits. |
| Evoluno | This app aims to increase personal development and resilience. A connection with a coach or a psychologist is proposed. |
| *goalmap: objectifs bien etre* | This app helps with personal development, defining personal goals, developing good habits and fighting procrastination. |
| LiveWell – Your health partner | This app is dedicated to wellness and brings together resources and tools to help the user achieve their health goals, whether it's through exercise tips, nutritious recipes, better sleep hygiene or managing their finances more effectively. The app allows the connection with specialists. |
| Mental Booster | This app aims to boost motivation and psychic performance while reprogramming the subconscious. |
| *Reflexe reussite* | This app aims to advise, offer self-coaching and stimulate motivation and personal development. A community linked to the app is proposed. The app allows the connection with specialists. |
| Sanvello | This app aims to help the user feel better, through therapy, coaching, coping techniques, meditations and goal and mood tracking. |
| *Soutien psy avec Mon Sherpa* | This app is a chatbot, a conversational agent, which will dialogue with the user to understand his mental health. |
| Teale | This holistic mental health app offers videos and self-care tools. It allows the user to talk to specialists. |
| *VOS – journal de l'humeur* | This app aims to build a daily mental balance through artificial intelligence and personalized activities. |

**Table S3.** Targets of the 12 mental health mobile apps included in the study

|  | ***Alan Mind: thérapie bien-être*** | ***Coach et Moi*** | ***Envol – Pleine santé*** | **Evoluno** | ***goalmap: objectifs bien etre*** | **LiveWell – Your health partner** | **Mental Booster** | ***Reflexe reussite*** | **Sanvello** | ***Soutien psy avec Mon Sherpa*** | **Teale** | ***VOS – journal de l'humeur*** |
| --- | --- | --- | --- | --- | --- | --- | --- | --- | --- | --- | --- | --- |
| Increase Happiness/Well-being | X | X | X | X | X | X | X | X | X | X | X | X |
| Mindfulness/Meditation/Relaxation | X | X | X | X | X | X | X | X | X | X | X | X |
| Reduce negative emotions | X | X | X | X | X | X | X | X | X | X | X | X |
| Anxiety/Stress | X | X | X | X | X | X | X | X | X | X | X | X |
| Anger | X | X | X | X | X | X | X | X | X | X | X | X |
| Behavior Change | X | X | X | X | X | X | X | X | X | X | X | X |
| Alcohol /Substance Use |  |  |  |  |  | X |  |  |  |  | X |  |
| Goal Setting | X | X | X | X | X | X | X | X | X | X | X | X |
| Relationships |  |  |  |  |  | X |  |  |  |  |  | X |
| Physical health |  |  |  |  | X | X |  |  |  |  |  |  |

**Table S4.** Theoretical background and strategies of the 12 mental health mobile apps included in the study.

|  | ***Alan Mind: thérapie bien-être*** | ***Coach et Moi*** | ***Envol – Pleine santé*** | **Evoluno** | ***goalmap: objectifs bien etre*** | **LiveWell – Your health partner** | **Mental Booster** | ***Reflexe reussite*** | **Sanvello** | ***Soutien psy avec Mon Sherpa*** | **Teale** | ***VOS – journal de l'humeur*** |
| --- | --- | --- | --- | --- | --- | --- | --- | --- | --- | --- | --- | --- |
| Assessment | X | X | X | X | X | X | X | X | X | X | X | X |
| Feedback |  |  |  |  |  |  |  |  |  |  |  |  |
| Information/Education | X | X | X | X | X | X | X | X | X | X | X | X |
| Monitoring/Tracking | X | X | X | X | X | X | X | X | X |  | X | X |
| Goal setting | X | X | X | X | X | X | X | X | X | X | X | X |
| Advice /Tips /Strategies /Skills training | X | X | X | X | X | X | X | X | X | X | X | X |
| CBT - Behavioural (positive events) | X | X | X | X | X | X | X | X | X | X | X | X |
| CBT – Cognitive (thought challenging) | X | X | X | X | X | X | X | X | X | X | X | X |
| ACT - Acceptance commitment therapy | X | X | X | X | X | X | X | X | X | X | X | X |
| Mindfulness/Meditation | X | X | X | X | X | X | X | X | X | X | X | X |
| Relaxation | X | X | X | X | X | X | X | X | X | X | X | X |

**Table S5.** Age group of the 12 mental health mobile apps included in the study.

|  | ***Alan Mind: thérapie bien-être*** | ***Coach et Moi*** | ***Envol – Pleine santé*** | **Evoluno** | ***goalmap: objectifs bien etre*** | **LiveWell – Your health partner** | **Mental Booster** | ***Reflexe reussite*** | **Sanvello** | ***Soutien psy avec Mon Sherpa*** | **Teale** | ***VOS – journal de l'humeur*** |
| --- | --- | --- | --- | --- | --- | --- | --- | --- | --- | --- | --- | --- |
| Children (under 12) |  |  |  |  |  |  |  |  |  |  |  |  |
| Adolescents (13-17) |  |  |  |  |  |  |  |  |  |  |  |  |
| Young Adults (18-25) | X | X | X | X | X | X | X | X | X | X | X | X |
| Adults | X | X | X | X | X | X | X | X | X | X | X | X |
| General |  |  |  |  |  |  |  |  |  |  |  |  |

**Table S6.** Technical aspects of the 12 mental health mobile apps included in the study.

|  | ***Alan Mind: thérapie bien-être*** | ***Coach et Moi*** | ***Envol – Pleine santé*** | **Evoluno** | ***goalmap: objectifs bien etre*** | **LiveWell – Your health partner** | **Mental Booster** | ***Reflexe reussite*** | **Sanvello** | ***Soutien psy avec Mon Sherpa*** | **Teale** | ***VOS – journal de l'humeur*** |
| --- | --- | --- | --- | --- | --- | --- | --- | --- | --- | --- | --- | --- |
| Allows sharing (Facebook, Twitter, etc.) | X | X |  |  | X |  | X |  | X |  |  | X |
| Has an app community | X |  |  |  | X |  |  | X | X |  |  | X |
| Allows password-protection | X | X | X | X | X | X | X | X | X | X | X | X |
| Requires login | X | X | X | X | X | X | X | X | X | X | X | X |
| Sends reminders | X | X | X | X | X | X | X | X | X | X | X | X |
| Needs web access to function | X | X | X | X | X | X | X | X | X | X | X | X |
