# Supplementary material for: Mental Health Mobile Apps in the French App Store: Assessment Study of Functionality and Quality
Source: JMIR Mhealth Uhealth. 2022 Oct 12;10(10):e41282. doi: 10.2196/41282 (PMC9607929; doi:10.2196/41282)
Supplement: Multimedia Appendix 3 [file mhealth_v10i10e41282_app3.docx]

**Multimedia Appendix 3: Mobile App Rating Scale-French (MARS-F) scoring**

**Table S1. Scoring by section**

| App Name | Data | Section A | Section B | Section C | Section D | Section ABCD | Section E | Section F |
| --- | --- | --- | --- | --- | --- | --- | --- | --- |
| *Alan mind : thérapie bien-être* | Mean | 2.82 | 3.78 | 3.48 | 3.04 | 3.22 | 1.72 | 2.67 |
|  | SD | 0.80 | 0.65 | 0.69 | 0.61 | 0.56 | 0.64 | 0.72 |
| *Coach et Moi* | Mean | 3.38 | 3.78 | 3.29 | 3.42 | 3.47 | 2.28 | 2.89 |
|  | SD | 0.56 | 0.95 | 0.56 | 0.55 | 0.55 | 1.01 | 0.95 |
| *Envol – Pleine santé* | Mean | 3.20 | 3.78 | 3.48 | 2.78 | 3.23 | 2.06 | 2.33 |
|  | SD | 0.88 | 1.00 | 1.12 | 1.18 | 0.97 | 1.23 | 1.19 |
| Evoluno | Mean | 3.38 | 3.97 | 3.70 | 3.31 | 3.54 | 2.42 | 3.31 |
|  | SD | 0.83 | 0.70 | 0.59 | 0.90 | 0.72 | 1.00 | 1.22 |
| *goalmap: objectifs bien etre* | Mean | 2.60 | 3.63 | 3.33 | 2.64 | 2.97 | 1.39 | 2.00 |
|  | SD | 0.71 | 0.67 | 0.74 | 0.41 | 0.49 | 0.39 | 0.78 |
| Livewell – Your health partner | Mean | 3.04 | 3.72 | 3.33 | 3.17 | 3.28 | 1.75 | 2.78 |
|  | SD | 0.68 | 0.94 | 0.74 | 0.74 | 0.73 | 0.90 | 1.12 |
| Mental Booster | Mean | 2.58 | 3.11 | 2.52 | 2.00 | 2.49 | 1.25 | 1.56 |
|  | SD | 0.59 | 0.71 | 0.62 | 0.75 | 0.61 | 0.33 | 0.97 |
| *Reflexe reussite* | Mean | 2.33 | 3.33 | 2.96 | 2.13 | 2.59 | 1.25 | 1.57 |
|  | SD | 0.69 | 0.74 | 0.59 | 0.72 | 0.61 | 0.33 | 0.76 |
| Sanvello | Mean | 3.04 | 3.56 | 3.59 | 2.85 | 3.18 | 1.47 | 2.17 |
|  | SD | 0.54 | 0.84 | 0.86 | 0.67 | 0.54 | 0.42 | 1.08 |
| *Soutien psy avec Mon Sherpa* | Mean | 3.80 | 4.47 | 3.89 | 3.46 | 3.85 | 2.69 | 2.59 |
|  | SD | 0.61 | 0.53 | 0.69 | 0.77 | 0.48 | 0.84 | 0.92 |
| Teale | Mean | 3.49 | 4.03 | 3.85 | 3.09 | 3.53 | 2.53 | 3.20 |
|  | SD | 0.79 | 0.29 | 0.78 | 0.52 | 0.47 | 0.91 | 0.90 |
| *VOS – journal de l’humeur* | Mean | 2.56 | 2.89 | 2.85 | 2.17 | 2.55 | 1.22 | 1.65 |
|  | SD | 0.75 | 0.63 | 0.88 | 0.81 | 0.71 | 0.26 | 1.02 |

**Table S2. Scoring by items**

| App Name | Data | Section A | | | | | Section B | | | | Section C | | | Section D | | | | | | |
| --- | --- | --- | --- | --- | --- | --- | --- | --- | --- | --- | --- | --- | --- | --- | --- | --- | --- | --- | --- | --- |
|  |  | Item 1 | Item 2 | Item 3 | Item 4 | Item 5 | Item 6 | Item 7 | Item 8 | Item 9 | Item 10 | Item 11 | Item 12 | Item 13 | Item 14 | Item 15 | Item 16 | Item 17 | Item 18 | Item 19 |
| *Alan mind : thérapie bien-être* | Mean | 2.44 | 2.78 | 2.78 | 2.44 | 3.67 | 3.89 | 3.78 | 3.78 | 3.67 | 3.56 | 3.44 | 3.44 | 3.22 | 2.78 | 3.44 | 3.22 | 3.89 | 1.67 | X |
|  | SD | 1.24 | 1.30 | 0.97 | 0.88 | 0.50 | 0.78 | 0.83 | 0.83 | 0.71 | 0.88 | 0.73 | 0.73 | 0.97 | 0.83 | 0.53 | 0.83 | 0.60 | 0.87 | X |
| *Coach et Moi* | Mean | 3.56 | 3.67 | 3.11 | 3.00 | 3.56 | 3.67 | 3.78 | 3.56 | 4.11 | 3.22 | 3.33 | 3.33 | 3.56 | 2.78 | 3.56 | 3.89 | 4.00 | 2.78 | X |
|  | SD | 0.73 | 0.50 | 0.6 | 1.00 | 1.01 | 1.00 | 0.83 | 1.74 | 1.05 | 1.09 | 0.50 | 0.50 | 0.73 | 0.44 | 0.88 | 0.93 | 0.71 | 0.83 | X |
| *Envol – Pleine santé* | Mean | 3.33 | 3.33 | 2.89 | 3.00 | 3.44 | 4.22 | 3.56 | 3.56 | 3.78 | 3.44 | 3.56 | 3.44 | 3.00 | 3.00 | 3.11 | 2.67 | 3.11 | 1.78 | X |
|  | SD | 1.32 | 1.22 | 0.60 | 1.00 | 1.24 | 0.67 | 1.33 | 1.51 | 0.83 | 1.33 | 1.13 | 1.24 | 1.22 | 1.32 | 1.27 | 1.41 | 1.45 | 1.20 | X |
| Evoluno | Mean | 3.44 | 3.33 | 3.22 | 3.00 | 3.89 | 3.67 | 3.89 | 4.22 | 4.11 | 4.00 | 3.78 | 3.33 | 3.89 | 2.78 | 3.78 | 3.67 | 3.44 | 2.33 | X |
|  | SD | 1.24 | 1.12 | 0.83 | 1.12 | 0.78 | 1.12 | 0.60 | 0.83 | 0.93 | 0.71 | 0.67 | 0.71 | 0.78 | 0.83 | 1.30 | 1.32 | 1.13 | 1.12 | X |
| *goalmap: objectifs bien etre* | Mean | 2.78 | 2.56 | 2.44 | 2.44 | 2.78 | 3.67 | 3.78 | 3.56 | 3.56 | 3.67 | 3.22 | 3.11 | 2.67 | 2.67 | 2.89 | 2.67 | 3.44 | 1.56 | X |
|  | SD | 0.83 | 1.01 | 1.01 | 0.53 | 0.97 | 0.71 | 0.83 | 0.88 | 0.73 | 0.71 | 0.83 | 0.93 | 0.87 | 0.71 | 0.60 | 0.50 | 0.53 | 0.73 | X |
| Livewell – Your health partner | Mean | 2.56 | 3.00 | 3.44 | 3.33 | 2.89 | 4.22 | 3.22 | 3.44 | 4.00 | 3.44 | 3.44 | 3.11 | 3.22 | 3.78 | 3.44 | 3.67 | 3.11 | 1.78 | X |
|  | SD | 0.73 | 1.22 | 0.73 | 0.87 | 0.60 | 0.83 | 1.39 | 1.13 | 1.00 | 1.01 | 0.73 | 0.93 | 0.97 | 0.67 | 1.13 | 1.12 | 1.17 | 0.97 | X |
| Mental Booster | Mean | 2.56 | 2.56 | 2.78 | 2.78 | 2.22 | 2.78 | 3.11 | 3.22 | 3.33 | 2.78 | 2.44 | 2.33 | 2.22 | 1.78 | 2.22 | 2.67 | 2.00 | 1.11 | X |
|  | SD | 0.88 | 0.88 | 0.67 | 0.67 | 0.44 | 1.09 | 0.93 | 1.09 | 0.50 | 0.97 | 0.53 | 0.50 | 1.09 | 0.67 | 1.09 | 1.12 | 1.22 | 0.33 | X |
| Reflexe reussite | Mean | 2.00 | 1.89 | 2.89 | 2.44 | 2.44 | 3.44 | 2.89 | 3.56 | 3.78 | 3.22 | 3.00 | 2.67 | 2.56 | 2.00 | 2.67 | 2.56 | 2.00 | 1.00 | X |
|  | SD | 1.00 | 0.78 | 0.78 | 0.53 | 1.33 | 0.73 | 1.27 | 1.09 | 1.09 | 0.83 | 0.71 | 0.71 | 0.88 | 1.12 | 1.00 | 0.73 | 1.00 | 0.00 | X |
| Sanvello | Mean | 3.00 | 3.33 | 2.78 | 2.78 | 3.33 | 3.89 | 3.33 | 3.56 | 3.44 | 3.56 | 3.89 | 3.33 | 3.22 | 2.56 | 3.00 | 3.11 | 3.56 | 1.67 | X |
|  | SD | 0.87 | 0.50 | 0.67 | 0.83 | 0.71 | 1.05 | 0.87 | 1.01 | 1.01 | 1.13 | 1.05 | 0.71 | 1.20 | 1.13 | 1.00 | 1.17 | 0.73 | 0.87 | X |
| *Soutien psy avec Mon Sherpa* | Mean | 3.78 | 4.11 | 3.22 | 3.89 | 4.00 | 4.56 | 4.33 | 4.33 | 4.67 | 4.33 | 3.67 | 3.67 | 3.67 | 2.78 | 3.56 | 3.89 | 4.11 | 2.78 | X |
|  | SD | 0.83 | 0.60 | 0.97 | 0.93 | 0.87 | 0.53 | 0.87 | 0.87 | 0.50 | 0.50 | 0.87 | 0.87 | 1.12 | 1.48 | 1.13 | 0.78 | 0.78 | 1.30 | X |
| Teale | Mean | 3.67 | 3.89 | 3.22 | 2.78 | 3.89 | 3.78 | 4.56 | 4.22 | 3.56 | 4.44 | 3.78 | 3.33 | 2.89 | 2.67 | 3.44 | 3.44 | 3.56 | 2.56 | X |
|  | SD | 1.22 | 1.05 | 0.67 | 0.67 | 1.17 | 0.67 | 0.53 | 0.44 | 0.73 | 0.73 | 0.97 | 1.00 | 1.17 | 0.50 | 1.01 | 1.01 | 1.01 | 0.73 | X |
| *VOS – journal de l’humeur* | Mean | 2.67 | 2.56 | 2.56 | 2.56 | 2.44 | 3.00 | 2.67 | 2.44 | 3.44 | 2.78 | 2.78 | 3.00 | 2.78 | 1.89 | 2.22 | 2.00 | 2.56 | 1.56 | X |
|  | SD | 0.87 | 0.88 | 0.88 | 1.24 | 0.88 | 0.87 | 0.50 | 1.01 | 0.88 | 1.09 | 1.30 | 0.71 | 0.97 | 1.05 | 0.97 | 1.12 | 1.24 | 0.88 | X |

| App Name | Data | Section E | | | | Mobile app specificities | | | | | |
| --- | --- | --- | --- | --- | --- | --- | --- | --- | --- | --- | --- |
|  |  | Item 20 | Item 21 | Item 22 | Item 23 | Awareness | Knowledge | Attitudes | Intention to  change | Help  seeking | Behaviour  Change |
| *Alan mind : thérapie bien-être* | Mean | 1.67 | 1.56 | 1.22 | 2.44 | 3.11 | 2.89 | 2.33 | 2.78 | 2.44 | 2.44 |
|  | SD | 0.71 | 1.01 | 0.67 | 0.53 | 1.17 | 1.17 | 0.87 | 0.67 | 0.73 | 0.73 |
| *Coach et Moi* | Mean | 2.44 | 2.56 | 1.22 | 2.89 | 2.78 | 2.89 | 2.89 | 2.89 | 3.22 | 2.67 |
|  | SD | 1.13 | 1.51 | 0.67 | 1.27 | 0.97 | 1.17 | 1.05 | 1.05 | 1.3 | 1.00 |
| *Envol – Pleine santé* | Mean | 2.00 | 2.11 | 1.44 | 2.67 | 2.22 | 1.78 | 2.56 | 2.44 | 2.67 | 2.33 |
|  | SD | 1.41 | 1.69 | 0.88 | 1.41 | 1.2 | 0.83 | 1.51 | 1.59 | 1.58 | 1.50 |
| Evoluno | Mean | 2.67 | 2.56 | 1.44 | 3.00 | 3.56 | 3.33 | 3.33 | 3.22 | 3.22 | 3.22 |
|  | SD | 1.22 | 1.01 | 0.88 | 1.22 | 1.24 | 1.32 | 1.22 | 1.3 | 1.39 | 1.30 |
| *goalmap: objectifs bien etre* | Mean | 1.44 | 1.33 | 1.00 | 1.78 | 1.78 | 1.89 | 2.00 | 2.11 | 2.44 | 1.78 |
|  | SD | 0.73 | 0.71 | 0.00 | 0.44 | 0.83 | 1.05 | 0.71 | 1.05 | 1.33 | 0.83 |
| Livewell – Your health partner | Mean | 1.67 | 1.67 | 1.22 | 2.44 | 3.00 | 2.89 | 2.78 | 2.44 | 2.89 | 2.67 |
|  | SD | 1.12 | 1.12 | 0.67 | 1.13 | 1.32 | 1.45 | 1.39 | 1.33 | 1.62 | 1.22 |
| Mental Booster | Mean | 1.22 | 1.11 | 1.00 | 1.67 | 1.44 | 1.44 | 1.56 | 1.56 | 1.78 | 1.56 |
|  | SD | 0.44 | 0.33 | 0.00 | 0.71 | 1.01 | 1.01 | 1.13 | 1.01 | 1.56 | 1.01 |
| Reflexe reussite | Mean | 1.33 | 1.00 | 1.00 | 1.67 | 1.56 | 1.44 | 1.56 | 1.44 | 1.89 | 1.56 |
|  | SD | 0.71 | 0.00 | 0.00 | 0.71 | 0.73 | 0.53 | 0.73 | 1.01 | 1.36 | 0.73 |
| Sanvello | Mean | 1.67 | 1.00 | 1.00 | 2.22 | 2.11 | 2.33 | 2.22 | 2.11 | 2.22 | 2.00 |
|  | SD | 0.71 | 0.00 | 0.00 | 1.09 | 1.17 | 1.22 | 1.09 | 0.93 | 1.39 | 1.00 |
| *Soutien psy avec Mon Sherpa* | Mean | 3.22 | 2.67 | 1.22 | 3.67 | 3.56 | 3.56 | 3.56 | 3.56 | 3.67 | 3.67 |
|  | SD | 0.83 | 1.32 | 0.67 | 1.00 | 0.88 | 0.88 | 1.01 | 1.24 | 1.00 | 0.87 |
| Teale | Mean | 2.56 | 2.67 | 1.67 | 3.22 | 3.00 | 3.00 | 3.44 | 3.44 | 3.33 | 3.00 |
|  | SD | 1.01 | 1.12 | 1.00 | 1.09 | 1.00 | 1.12 | 1.01 | 1.01 | 1.12 | 0.87 |
| *VOS – journal de l’humeur* | Mean | 1.11 | 1.00 | 1.00 | 1.78 | 1.89 | 1.33 | 1.67 | 1.56 | 2.00 | 1.44 |
|  | SD | 0.33 | 0.00 | 0.00 | 0.83 | 1.27 | 0.71 | 1.32 | 1.13 | 1.58 | 0.88 |
